# Knowledge, attitudes and practices of general medical practitioners in developed countries regarding oral cancer: an integrative review

**DOI:** 10.1093/fampra/cmaa026

**Published:** 2020-04-07

**Authors:** Nidhi Saraswat, Bronwyn Everett, Rona Pillay, Neeta Prabhu, Ajesh George

**Affiliations:** 1 Centre for Oral Health Outcomes and Research Translation, School of Nursing and Midwifery, Western Sydney University/South Western Sydney Local Health District/Ingham Institute for Applied Medical Research, Liverpool, NSW; 2 School of Nursing and Midwifery, Western Sydney University, Parramatta, NSW; 3 Westmead Centre for Oral Health. Dentistry, University of Sydney, Sydney, NSW, Australia; 4 School of Dentistry, Faculty of Medicine and Health, University of Sydney, NSW, Australia

**Keywords:** Attitudes, general practitioners, knowledge, oral cancer, physicians, practices

## Abstract

**Background:**

Oral cancer is a public health concern and is widespread in developing countries, particularly in South Asia. However, oral cancer cases are also rising in developed nations due to various factors, including smoking, viruses and increased migration from South Asia. In this context, the role of general medical practitioners (GPs) in identifying oral cancer is becoming increasingly important and, while some studies have explored their perspective about oral cancer, a synthesis of these results has not been undertaken.

**Objective:**

The objective of this integrative review is to synthesize existing evidence regarding oral cancer-related knowledge, attitudes and practices of GPs in developed countries.

**Methods:**

Four electronic databases were searched to identify studies focussing on the objective of this review. The inclusion criteria were: peer-reviewed English language publications; studies conducted in developed countries involving GPs; explored at least one study outcome (knowledge/attitudes/practices). No restrictions were placed on the publication date.

**Results:**

A total of 21 studies involving 3409 GPs were reviewed. Most studies revealed limited knowledge of GPs about emerging risk factors, such as betel nut chewing (0.8–50%). Significant variation (7–70%) was evident in routine oral examination practices of GPs. Most GPs felt unsure about diagnosing oral cancer and many (38–94%) raised the need for further education. No study explored the specific relevance of GPs’ practices concerning South Asian immigrants.

**Conclusion:**

This review suggests the need for educational programs to enhance GPs’ knowledge regarding oral cancer. Further research exploring oral cancer-related practices of GPs caring for South Asian immigrants is warranted.

**Key Messages**

- Poor knowledge of emerging oral cancer risk factors among general medical practitioners (GPs).
- Lack of confidence and limited oral cancer screening practices among GPs.
- Need for oral cancer-related education and training for GPs.
- Further research required in other developed countries due to migration patterns.

## Background

Oral cancer is a growing public health problem worldwide. This non-communicable disease is one of the leading causes of death in some Asia-Pacific countries (1,2) and is among the top 15 most common cancers in the world (3,4). A total of 354 864 lip and oral cavity cancer cases were estimated worldwide in 2018 constituting 2% of all new cancer cases (3). Oral cancer contributes 1.9% to world cancer mortality rates despite the wide variation in its incidence across the globe (1,5). While this type of malignancy is more widespread in South Asia (3), it has also become a matter of concern in developed nations as well (6,7). Over the past decade, there has been an increase in oral cancer rates of developed countries, such as the USA (8), Australia (9), UK (10,11) and some other parts of Europe (12), adding to the economic burden in terms of health expenditure in these countries (13–18).

A myriad of factors is responsible for the aggressive nature of oral cancer worldwide. These include chronic smoking, frequent use of smokeless tobacco/areca nut/betel quid, alcohol consumption, radiation, viruses, poor oral hygiene and genetic factors (1,19). Further, oral cancer is more prevalent in men and older-aged people and frequently common among lower socio-economic groups (1, 20). Oral cancer incidence related to human papilloma virus (HPV) infections has also increased in some developed countries (21). The contribution of these risk factors to the oral cancer burden varies globally; for instance, smoking is responsible for approximately 71% of the deaths from oral cancer in high-income countries, while 37% in low-income and middle-income countries (21). There have also been reports suggesting increased migration as a contributing factor (22,23) to the rise of oral cancer in developed countries with studies exploring the potential association of risk practices of South Asian immigrants and oral cancer rates in countries like the USA (24–28), UK (29–32) and European countries (33).

In contrast to other malignancies, oral cancer is considered to be a more serious health issue due to its low 5-year survival rate, largely attributable to delayed diagnosis due to the asymptomatic nature of the condition in the early stages (34,35). Another contributing reason behind late identification of oral cancer is lack of accessible and affordable dental referral pathways in many countries (36), which often results in complex, invasive and expensive therapeutics (35,37). Thus, early identification and prompt referrals can potentially improve outcomes and prognosis, leading to higher survival rates (36).

Early diagnosis is crucial for reducing overall oral cancer morbidity. Although dentists have a definitive role in diagnosing oral cancer (38), the critical role of general medical practitioners (GPs) in early identification of such neoplasms cannot be underestimated (39). GPs are the most commonly sought primary health care provider and patients are more likely to visit GPs compared to dentists (40,41). This is particularly relevant in developed countries, which generally have well-structured, accessible and affordable health care systems (42,43). Further, the high cost of dental treatment also deters patients from visiting dentists regularly (44). Hence, it becomes even more pivotal to ensure that GPs have adequate knowledge and awareness of oral cancers.

In light of the growing emphasis on the role of GPs in early identification of oral cancer, some studies have been undertaken to assess their perspective and practices concerning oral cancer risk (35,40,41,45–51). However, a synthesis of these results has not yet been undertaken. This integrative review aims to synthesize all available evidence regarding the knowledge, attitudes and practices of GPs regarding oral cancer in developed countries.

## Methods

This integrative review used the Preferred Reporting Items for Systematic Reviews and Meta-Analyses (PRISMA) statement (52) to report the findings. The protocol for this review was submitted to PROSPERO—International Prospective Register of Systematic Reviews (CRD42019146969). The integrative review approach allows the combination of diverse methodologies, including qualitative, quantitative and mixed-method studies, to gain better insights into the research area.

### Eligibility criteria

Studies were included provided they met the following criteria: (i) peer-reviewed publications in the English language; (ii) conducted with GPs in developed and high-income countries and (iii) explored at least one study outcome (knowledge, attitudes or practices associated with oral cancer risk). All qualitative, quantitative, and mixed-method designs were eligible. No restrictions were placed on the year of publication.

### Data sources and search strategy

A search of the four electronic databases Ovid-Medline All, CINAHL, Scopus and ProQuest Central was undertaken using Medical Subject Headings (MeSH) terms and synonyms including oral cancer, mouth neoplasms, general practitioners, primary health care providers, physicians, doctors, health professionals, developed countries, knowledge, perception and awareness. These terms were used in combination using ‘Boolean’ operators (AND/OR). The filter applied in the search included language (English). A university librarian experienced in undertaking literature reviews was also consulted to ensure the relevance of individual search strategies. The reference lists of selected articles chosen to be included in the review were explored to ensure that relevant studies were not missed. A detailed search strategy is included in Supplementary file 1 indicating the keywords used for the literature search.

### Study selection

**Figure 1.**
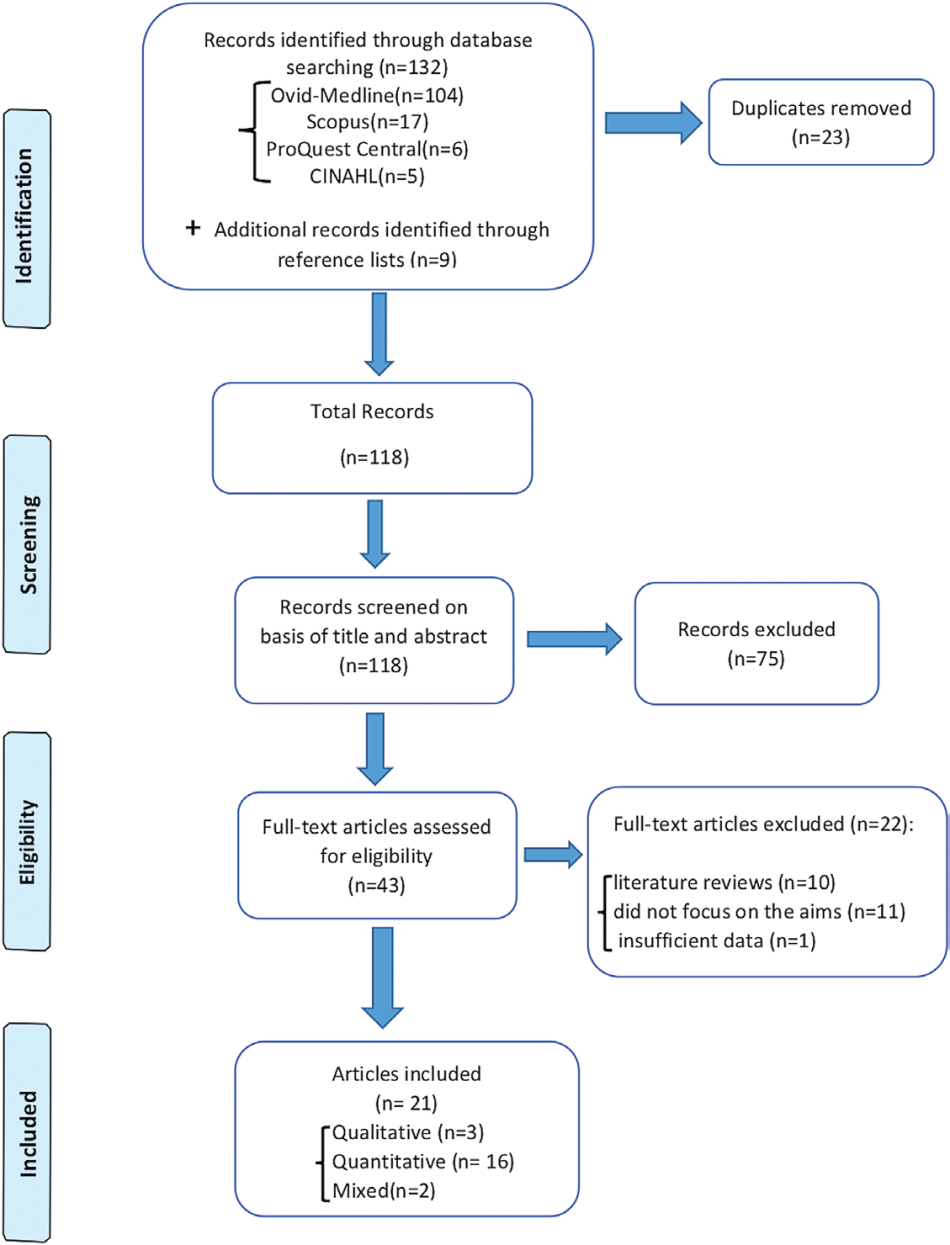
Study selection process.

The search results were organized using EndNote bibliographic software and duplicates were removed. Two experienced authors (NS and RP) independently assessed the suitability of extracted studies by screening title and abstract as per the inclusion criteria. Thereafter, the full text of selected articles was reviewed by two authors (NS and RP) independently and, then, together in case of doubt or discrepancy. This process of full-text screening has been explained in Supplementary file 2. A third author (AG) was consulted to resolve any discrepancies in judgement regarding the inclusion of articles. The screening and selection process has been illustrated in Figure 1 (study selection process).

### Quality assessment

The critical appraisal for all the selected articles was undertaken independently by two reviewers (NS and RP) to assess the methodological quality. Two separate quality checklists tools were used—Critical Appraisal Skills Programme (CASP) checklist for qualitative studies (53) and the Joanna Briggs Institute (JBI) checklist for analytical cross-sectional studies (54). Both of these tools have been commonly used for assessing qualitative and cross-sectional studies (55). A third reviewer (AG) was referred for the final decision in case of differences in quality assessments. The quality of these studies was calculated using scoring criteria (56). The score was given as a percentage (1 point for each applicable item) and the overall quality was rated as good (80–100%), fair (50–79%) and poor (<50%) (56). The critical appraisal of the studies is provided in Supplementary file 3.

### Data extraction

The data extraction form (see Supplementary file 4) was developed independently by two authors (NS and RP) and modified as required. The data extraction tables (see Tables 1 and 2) comprised information regarding author, year of publication, country, study characteristics and key outcomes. These tables were further checked by two other authors (RP and AG) for accuracy.

**Table 1.** Characteristics of included studies (dated 1995–2018)

S.N.	Author and year of publication	Country	Methodology; data collection method	Sample size (GPs)	Sample characteristics	Response rate (%)		
					Age (in years)	Gender (%)	Years of experience (range)	
1	Yellowitz *et al.* 1995 (45)	USA	Quantitative survey (questionnaire)	93	20–79	M = 88; F = 12	NR	78.8
2	McCunniff 2000 (49)	USA	Quantitative survey (questionnaire)	110	NR	M	NR	NR
3	Greenwood and Lowry 2001 (61)	UK	Quantitative survey (questionnaire)	151	NR	NR	8–31	71.9
4	Canto *et al.* 2002 (46)	USA	Quantitative survey (questionnaire)	240	NR	M = 58; F = 42	4–32	35.4
5	Canto *et al.* 2002 (47)	USA	Qualitative one focus group with 10 GPs + face-to-face interviews with 9 GPs	19	NR	NR	NR	NR
6	Macpherson *et al.* 2003 (68)	Scotland (UK)	Mixed-method interviews + questionnaire (face-to-face, semi- structured interviews of 11 GPs + survey of 198 GPs)	209	NR	M = 56; F = 44	NR	57
7	Nicotera *et al.* 2003 (41)	Italy (Europe)	Quantitative survey (questionnaire)	189	Mean age = 51	M = 64.4; F = 35.6	NR	38.8
8	Sohn *et al.* 2005 (50)	USA	Quantitative survey (questionnaire)	79	29–60	M = 39.5; F = 60.5	NR	56.4
9	Patton *et al.* 2006 (35)	USA	Quantitative survey (questionnaire)	273	Mean age > 40	M = 67.9; F = 32.1	NR	25.8
10	Cruz *et al.* 2007 (51)	USA	Qualitative interviews (face-to-face, structured interviews)	4	NR	NR	NR	70^a^
11	Carter and Ogden 2007 (40)	UK	Quantitative survey (questionnaire)	238	NR	NR	NR	71.26
12	NiRiordain and McCreary 2009 (64)	Ireland (Europe)	Quantitative survey (questionnaire)	236	NR	M = 61.9; F = 38.1	4–57	52.2
13	Applebaum *et al.* 2009 (38)	USA	Quantitative survey (questionnaire)	118	NR	M = 53; F = 47	7–65	25.8
14	Reed *et al.* 2010 (65)	USA	Quantitative survey (questionnaire)	165	40–59	M = 100	23 (average)	43
15	Morse *et al.* 2011 (60)	Puerto Rico^b^	Qualitative interviews (face-to-face, key- informant interviews)	2	NR	NR	NR	90.9^a^
16	Ismail *et al.* 2012 (67)	USA	Mixed-method survey (pre-questionnaire)	274	30–69	M = 64.6; F = 35.1	NR	16.7
17	Hertrampf *et al.* 2014 (62)	Germany (Europe)	Quantitative survey (questionnaire)	327	30–69	M = 50; F = 47	NR	13^a^
18	Shanahan *et al.* 2018 (48)	Ireland (Europe)	Quantitative survey (questionnaire)	221	19–29	M = 34.8; F = 64.2	3–6^c^	5.2
19	Shimpi *et al.* 2018 (66)	USA	Quantitative survey (questionnaire)	43	25–70	M = 45.9^a^; F = 54.1^a^	NR	20^a^
20	Gelažius *et al.* 2018 (19)	Lithuania (Europe)	Quantitative survey (questionnaire)	42	Mean age = 52	M = 35.7; F = 64.3	NR	NR
21	Lechner *et al.* 2018 (63)	UK	Quantitative survey (questionnaire)	376	NR	M = 40.9; F = 59.1	NR	72.9

^a^Data reported for all the participants (multiple health professionals involved).

^b^Puerto Rico is unincorporated territory of the USA.

^c^Data reported for 77% of the participants.

**Table 2.** Findings and quality rating of the studies (dated 1995–2018)

S.N.	Author, year of publication and country	Method and sample size	Findings			Quality scores and quality rating^a^
			Knowledge	Attitudes	Practices	
1	Yellowitz *et al.* 1995 (45); USA	Questionnaire; *n* = 93	• 63.3% Responded that early detection of oral cancer lesions improves survival rates. • 92.6% considered patients (>45 years) at higher risk for oral cancer. • 55.8% could distinguish oral cancer lesions if any lesion was bigger than 3 mm size.	• 32.6% believed their knowledge to be current. • 20.9% strongly felt their knowledge not being up to date. • 84.1% strongly agreed about the importance of examining elderly patients for oral cancer. • 45% felt inadequately trained for oral cancer.	• 18% provided routine oral examination of 50% of their patients. • 7% indicated conducting complete examination of patients for oral cancer.	75%; B
2	McCunniff 2000 (49); USA	Questionnaire; *n* = 110	• Identified major risk factors as alcohol use (68%) and tobacco consumption (97%). • 77% mentioned floor of the mouth as a common site for oral cancer.		• 7% routinely examined oral cavity for oral cancer lesions.	50%; B
3	Greenwood and Lowry 2001 (61); UK	Questionnaire; *n* = 151	• Habits (e.g. Betel quid chewing) predisposing to oral cancer identified 50.3%. • 90.7% identified smoking as a risk factor and alcohol was identified as a cause by 45.7%. • 47.3% had knowledge of premalignant lesions. • All GPs mentioned more than one treatment modality.		• 68.2% were likely to examine all sites of mouth in relation to oral cancer. • 74.2% gave preference to OMFS for referrals, while ENT was a second priority for 24% in terms of referrals.	50%; B
4	Canto *et al.* 2002 (46); USA	Questionnaire; *n* = 240	• 83% knew oral cancer increases with age. • 89% identified alcohol as a risk factor. • More than 80% specified SCC as the commonest type of oral cancer. • 60% knew that oral cancer lesions are diagnosed at the advanced stages. • GPs who graduated before 1970 presented better knowledge scores.	• More recently graduated were less likely to agree that their oral cancer knowledge was current. • 12% rated oral cancer training as very good during medical school education. • 62% strongly agreed they were adequately trained to examine patients for oral cancer.	• More than 80% knew how to examine patients for oral cancer. • 77% used to ask about risk factors for oral cancer while taking medical history. • 15% said they provided an oral cancer examination of patients aged >40 years. • 32% never provided oral cancer examination to patients of age group 18–39 years.	37.5%; C
5	Canto *et al.* 2002 (47); USA	Focus groups and interviews; *n* = 19	• All unaware and surprised by high oral cancer mortality rates • Highly aware of major risk factors like tobacco and alcohol use	• Did not accept and trust the validity of the statistics • Believed that patients are more likely to see them more than dentists due to cost factor • Prioritized their role as gatekeeper in managed care during referrals • Agreed about raising oral cancer awareness among public and health professionals • Interested in receiving oral cancer education as part of CME	• Seen very few cases of oral cancer in their practices • Used standard practice to assess patients’ risk behaviours, such as tobacco and alcohol use • Generally, do not talk about risks of oral cancer • Often conduct risk assessment themselves rather than asking a nurse to do it • No one reported conducting routinely a comprehensive oral cancer examination • More likely to refer cases of suspicious lesions to ENT specialists	80%; A
6	Macpherson *et al.* 2003 (68); Scotland (UK)	Mixed method; *n* = 209	• High awareness about smoking (97%) as a risk factor for oral cancer but less for alcohol (79%) and age (76%) • 72% GP considered leukoplakia as a potentially malignant lesion. • 43% considered trauma as an important causative factor. • 20% mentioned fungal infections responsible for oral cancer. • 23% considered viruses as a relevant risk factor. • 26% underwent formal training for counselling regarding smoking. • 37% never received any organized tuition on oral cancer.	• 15% felt confident detecting of detecting malignant oral lesions. • 23% expressed confidence in their ability to assess urgent referrals of oral lesions. • All felt dentist as mouth specialist. • 66% felt that their role to be major in oral cancer detection. • 70% expressed confidence in giving advice to patients regarding oral cancer. • Wished for more training in oral cancer detection (91%) and prevention (79%) • Preferences for the format of training were not profession specific.	• Lack of training (70%) and lack of time (47%) were perceived as barriers to undertake oral cancer examination. • 94% indicated they examined patients’ mouth usually in response to complaint of soreness. • 81% conducted oral cavity examination as a result of knowledge of a pre-existing oral condition. • 57% stated to consider urgent referral in case of persistence of oral lesion more than 4–5 weeks. • 87% routinely made enquiries to patients in relation to smoking habits. • 67% wanted additional advice on referral pathways	37.5%; C
7	Nicotera *et al.* 2003 (41); Italy (Europe)	Questionnaire; *n* = 189	• 87.6% correctly indicated tobacco usage and 64% identified alcohol consumption as risk factor for oral cancer. • Some mentioned prior oral lesion (31.5%) and older age (2.8%) as risk factor. • 60.9% knew SCC as the most common form of oral cancer. • 25.8% mention both common sites (tongue and floor of mouth) for oral cancer. • Scientific journals (85.1%), continuing education courses (52.1%) and colleagues (16.5%) were reported as sources of information.	• 84.9% felt the need for additional information.	• 26.8% knew how to examine the tongue. • 63.8% routinely examined the oral cavity of patients. • 37.1% responded that they provided oral examination to patients older than 40 years. • Reported asking about tobacco use (85.1%) and alcohol consumption (82.5%).	50%; B
8	Sohn *et al.* 2005 (50); USA	Questionnaire; *n* = 79	• 44% were labelled as high knowledge score and 56% with low knowledge regarding oral cancer. • 45% were not aware of areas of the tongue more likely to develop oral cancer. • 50% mentioned the relevance of size of swelling to determine the staging of oral cancer. • 60% prioritized the role of bleeding in determining the diagnosis. • 85% learnt from mailings sent from professional medical organizations. • Preferred sources of information were CME, professional meetings, journal articles and educational mailings.	• 92% agreed that annual oral cancer examinations should be performed for adults aged ≥40 years. • 96% strongly agreed for an annual examination of tobacco users. • 48% agreed that oral cancer exams should be separate reimbursable procedures. • 76% felt the need for more oral cancer education through CME. • 51% felt the need for a referral system to increase oral cancer screening. • 45% were willing to participate in a network to promote early screening for oral cancer.	• 70% reported screening patients for oral cancer during routine examination. • More likely to be male physicians who regularly performed oral cancer screening. • 89% could correctly examine the tongue for oral cancer screening. • More than 50% considered the age of patients while screening patients for oral cancer. • Lack of adequate training (64%), shortage of specialists for referrals (48%) and lack of time (15%) were perceived as possible barriers for oral cancer examinations.	50%; B
9	Patton *et al.* 2006 (35); USA	Questionnaire; *n* = 273	• 92% stated that early detection of oral cancer improves 5-year survival rates. • 31% considered their oral cancer knowledge to be current.	• 61.2 felt confident and adequately trained to examine patients of oral cancer. • More confident (81%) in their training for tobacco cessation than alcohol cessation	• More than 90% usually asked patients about cancer history and the use of tobacco and alcohol.	62.5%; B
10	Cruz *et al.* 2007 (51); USA	Interviews; *n* = 4	• Described little understanding of oral cancer • Low awareness and less knowledge of signs, symptoms and risk factors • Received very little training on oral cancer in medical school	• Agreed on lack of appropriate access to health care in communities where they practice • All cited a lack of compliance by their patients. • Half agreed that oral cancer examination should be a shared responsibility.	• Mentioned communication difficulties as barriers for preventive regimens. • Half of the participants reported routinely checking for signs of oral cancer as general medical workup. • All routinely counsel their patients about smoking cessation. • All preferred dentists for referrals of oral lesions	80%; A
11	Carter and Ogden 2007 (40); UK	Questionnaire; *n* = 238	• 43.28% identified alcohol as a risk factor for oral cancer. • More than 90% knew about smoking as a cause of oral cancer. • Less than 10% knew about the association of betel nut with oral cancer. • Approx. 72% named ulceration as a major oral change associated with oral cancer. • No one mentioned erythroplakia linked with oral cancer.	• 25.2% felt that they had sufficient knowledge regarding the prevention and detection of oral cancer. • 71.4% requested further information/ training on oral cancer as a preferred format compared to meetings/seminars.	• 20.17% routinely examined patients’ oral mucosa. • 65.1% did not examine the oral mucosa of high-risk patients. • More than one-third reported regularly advising their patients on risk factors for oral cancer. • Preferred oral medicine and OMFS as their preferred points of referral.	25%; C
12	NiRiordain and McCreary 2009 (64); Ireland (Europe)	Questionnaire; *n* = 236	• Identified risk factors (mean number of factors = 1.9) for oral cancer. • Smoking (98.7%), alcohol (50.8%) and poor oral hygiene (20.7%) were identified as major risk factors. • 0.8% identified betel nut chewing as a causative factor. • Ulceration (67.4%) was identified as a major oral change associated with oral cancer. • 0.4% identified erythroplakia associated to be associated with oral cancer. • 89.4% denied the availability of any CME course in their region. • 21.6% received formal teaching on oral cavity examination during under graduation.	• 34.3% felt confident in their ability to detect oral malignancies. • Two-thirds were unsure about their ability to detect oral cancer. • 94% declared interest in further information or training on oral cancer.	• 65.3% reported regularly examining oral mucosa of the patients. • Preferred referrals to ENT (37.7%) and dentist (9.3%)	37.5%; C
13	Applebaum *et al.* 2009; USA (38)	Questionnaire; *n* = 118	• 96% mentioned eight risk factors for oral cancer correctly. • 99% correctly identified the use of tobacco as a high-risk factor. • Identification of SCC as the most common form of oral cancer. • More than 90% mentioned that early oral cancer lesions are asymptomatic. • 10% mentioned erythroplakia and leukoplakia to be associated with oral cancer. • More recently graduated expressed greater knowledge levels.	• 91% believed that dentists were more qualified to perform an oral examination. • 67% felt that physicians were qualified to perform the examination for oral cancer. • 46% believed that they were adequately trained to examine patients for oral cancer. • 5% strongly agreed that their oral cancer knowledge was current.	• Proficient in assessing the risk factors for oral cancer when taking the medical history of patients.	62.5%; A
14	Reed *et al.* 2010 (65); USA	Questionnaire; *n* = 165	• More than 88% identified tobacco use (smoking, chew or snuff) to be associated with oral cancer. • Alcohol use (>37%) and HPV (45%) were mentioned as medium risk for oral cancer. • More than half were aware of tobacco cessation sources.	• 38–58% were interested in receiving training in oral cancer screening. • 49% were interested in receiving training in tobacco cessation.	• 14–20% conducted a routine oral cancer examination. • 84–87% reported assisting patients regarding smoking counselling. • Older practitioners (>60 years) were more likely to provide oral cancer screening.	50%; B
15	Morse *et al.* 2011 (60); Puerto Rico*	Interviews; *n* = 2	• Most of the participants reported a lack of knowledge regarding early signs and symptoms of oral cancer. • Many identified some previous professional training related to oral cancer prevention, identification and treatment as part of a course. • Had not received any training in conducting oral cancer examinations • Acknowledged lack of coordination during referrals behind delayed diagnosis	• Felt impact of socio-economic status of patients on their wait prior to seeking health care • Felt delayed diagnosis largely attributed to bureaucratic complexities • Perceived dentist with greatest responsibility to provide oral cancer examinations • The majority of participants had concerns regarding the quality of oral cancer examinations.	• All reported conducting oral cancer examination only on high-risk patients or in case of patient’s specific concern for abnormality. • One stated conducting only visual oral examinations. • Considerable confusions regarding referral pathways.	90%; A
16	Ismail *et al.* 2012 (67); USA	Questionnaire; *n* = 274	• 21.3% identified the tongue as a common site for oral cancer. • 87.4% mentioned the correct sequence of examination and areas to be checked for oral cancer.		• 23% routinely screened patients for oral cancer. • 31.5% never conducted a routine oral cancer examination. • 43.9% usually screened patients (aged >40 years) for oral cancer.	75%; B
17	Hertrampf *et al.* 2014 (62); Germany (Europe)	Questionnaire; *n* = 327	• More than 90% knew about tobacco and alcohol use as a major risk factor for oral cancer. • 88–90% said early detection improves survival rates. • More than one-third identified SCC as the most common form of oral cancer. • Half of the participants identified the tongue as a common site. • More than 70% never attended any CME course regarding oral cancer.			62.5%; B
18	Shanahan *et al.* 2018 (48); Ireland (Europe)	Questionnaire; *n* = 221	• Tobacco smoking (94%), alcohol use (63%) and viral factors (29%) were identified as major risk factors. • Ulceration (67%), leukoplakia (35%), exophytosis (31%) and erythroplakia (15%) were considered to be associated with oral cancer.	• 60% felt unsure about diagnosing oral cancer based on clinical appearance. • 84% requested further education on oral cancer.	• 81% mentioned not conducting a routine examination of oral mucosa of patients. • 5% informed their patients of risk factors for oral cancer. • The majority indicated that they would prefer to provide referrals of suspected oral cancers to ENT surgeons (53%), while others preferred OMFS (42%).	50%; B
19	Shimpi *et al.* 2018 (66); USA	Questionnaire; *n* = 43	• All identified one or more symptoms of oral cancer correctly. • 98% could recognize the visual-based image of aphthous ulcer lesion. • Less than half could identify the tongue as the most common site for oral cancer. • 56% reported limited training on oral cancer examination.	• 52% felt comfortable in educating patients about oral cancer. • 61% perceived responsibility in screening patients for oral cancer annually. • 53% were comfortable in performing oral cancer screening.	• 78% indicated not performing oral cancer screening on all patients. • Practitioners with more than 10 years of experience were more comfortable performing oral cancer examination.	62.5%; B
20	Gelažius *et al.* 2018 (19); Lithuania (Europe)	Questionnaire; *n* = 42	• 61.3% reported low knowledge after university studies. • 83.3% reported minimal familiarity with oral cancer. • 16.7% mention the five most common carcinogens. • 69.9% could answer the most frequent type of oral cancer. • 33.3% picked up oral cancer symptoms correctly.	• 87.7% had patient’s carcinophobia during visitations. • 50.9% wanted to have an annual week of oral cancer prevention at their workplace. • 45.2% agreed that POCD should be an individual procedure done at primary appointments.	• 76.2% gives information to patients about the negative influence of tobacco. • 28.3% had dealt with patients with oral cancer. • 17% usually checked the oral cavity of patients.	25%; C
21	Lechner *et al.* 2018; UK (63)	Questionnaire; *n* = 376	• 19.4% rated their oral cancer knowledge very good. • 17.7% reported less knowledge about HPV- associated oral cancer. • More awareness about well-established risk factors like smoking (99.4%), chewing tobacco (96.6%) and alcohol use (94.3%) • 32.9% recognized oral cancer risk related to areca nut chewing.			50%; B

ENT, ear, nose and throat specialty; OMFS, oral and maxillofacial surgery; POCD, Primary Oral Cancer Diagnostics; SCC, squamous cell carcinoma.

^a^A or good quality (80–100%); B or fair quality (50–79%); C or poor quality (<50%).

^b^Puerto Rico is an unincorporated territory of the USA.

### Data synthesis

Since the studies to be included were heterogeneous, a meta-analysis was not possible for this review. Therefore, outcomes of all studies were reported through narrative synthesis. This unfolding narrative synthesis with connecting themes is more appropriate to ‘tell a story’ (57) than the comprehensive categorization of all the individual studies. It aims to provide a relatively complete picture regarding knowledge, attitudes and practices of GPs concerning oral cancer risk in this review.

### Definition of terms

For the purpose of this review, the following terms have been modified and used: ‘developed countries’ denotes nations that have developed economies with high income like the USA, UK, Canada, Australia and New Zealand (58). ‘Knowledge’ signifies one’s understanding and level of information regarding oral cancer risk (59). ‘Attitudes’ refers to one’s inclinations, perceptions and beliefs associated with the oral cancer risk (59). ‘Practices’ denotes the habits and actions of oral cancer identification and prevention (59).

## Results

The search of databases identified 132 records; 23 were duplicates and subsequently removed. An additional nine records were found through a manual search of reference lists of identified studies, which resulted in a total of 118 records. The process of initial screening based on title and abstract resulted in the exclusion of 75 articles, leaving 43 for full-text screening. After full-text review, a further 22 articles were excluded as they were literature reviews (*n* = 10), did not focus specifically on oral cancer-related knowledge, attitudes and practices (*n* = 11) and data regarding GPs could not be elicited from studies involving multiple health care providers (*n* = 1). This left 21 studies for inclusion in the review: 3 were qualitative (46,51,60), 16 were quantitative (19,35,38,40,41,45,47–50,61–66) and 2 were mixed-method designs (67,68) (see Fig. 1 for the study selection process).

### Study characteristics

The 21 studies included in this review were published between 1995 and 2018 and were conducted in the USA (*n* = 11), UK (*n* = 4), Europe (*n* = 5) and Puerto Rico (US-owned territory; *n* = 1). Sample sizes ranged from 2 (60) to 376 (63) with a total of 3409 GPs. The age of the participants ranged from 19 to 79 years and consisted of mostly males (see Table 1 for study characteristics).

The quality of the studies was rated as good (*n* = 3; score ≥ 80), fair (*n* = 13; score 50–79%) and poor (*n* = 5; score < 50%; see Table 2 for findings and quality rating of studies). Due to limited literature in this area, irrespective of their quality, all studies were included in this review to allow the reader to make their own judgement.

### Study findings

The narrative synthesis facilitated the categorization of the study findings into three domains.

#### Domain 1: oral cancer knowledge

All 21 studies explored the knowledge of GPs about oral cancer risk. These studies assessed the level of information and awareness of participants regarding oral cancer risk factors, diagnosis and treatment strategies. Most studies indicated sound knowledge among GPs about oral cancer causative factors like smoking (88–99.4%) (19,40,48,61,63–65,68) and tobacco use (87.6–99%) (19,38,41,48,49,62,65). However, considerable variability in knowledge levels was noted among participants regarding other risk factors, including alcohol consumption (37–94.3%) (19,40,41,47–49,61–65,68), viral infections (23–73.8%) (48,63,65,68), old age (2.8–83%) (41, 45, 47, 68) and betel nut/quid chewing (0.8–50%) (40, 61, 63, 64). The uncertainty regarding alcohol as a risk factor for oral cancer was also evident in a qualitative study (68):

‘Trauma, probably smoking, denture wear causing ulceration… I don’t know about the alcohol factor, although I see no reason why it shouldn’t be a factor as it affects your health in lots of other ways’. (p. 278) (68)

Other oral cancer risk-related factors correctly identified by GPs included prior oral lesions (31.5%) (41), trauma (43%) (68), fungal infections (20%) (68) and poor oral hygiene (20.7%) (64). GPs were generally knowledgeable about squamous cell carcinoma being the most common type of oral cancer (60.9–80%) (19,38,41,47,62) but were less sure about the most common sites for this cancer, such as floor of mouth (25.8–77%) (41,49,50,66) and tongue (21.3–55%) (41,50,66,67) or associated symptoms like ulceration (33.3–72%) (19,40,48,64) and premalignant lesions (10–72%)(19, 38, 40, 48, 61, 64, 68).

Participants had a mixed understanding (60–92%) about how early detection improves 5-year survival rates (35,45,47). Some studies though reported a lack of awareness (46,51) and limited understanding (46,60) among GPs concerning the prevalence of oral cancer. These findings were also reflected in the following statement:

 ‘Honestly, very poor [referring to early oral cancer detection in Puerto Rico] because realistically, it [oral cancer] is not discovered as much because people [health practitioners] do not perform oral exams on patients. They do not open their mouths. Sometimes people arrive with something they have had for months, and no one [checks the mouth]’ (p. 4) (60)

The main source of information regarding oral cancer for GPs were Continuing Medical Education/CME (10.6–52.1%) (41,62,64), professional meetings/colleagues (16.5%) (41), scientific journals (85.1%) (41) and professional mailings (85%) (50).

#### Domain 2: oral cancer attitudes

The attitudes of GPs towards oral cancer risk were reported in 16 studies. The attitude items were mainly related to the perception and inclination of participants towards oral cancer awareness. Few participants (5–32.6%) felt their oral cancer risk-related knowledge was current (38,40,45) and several GPs (38–94%) were interested in receiving further information and education on this topic (40,41,48,50,64,68). Some studies also revealed a lack of confidence (15–60%) (35,48,64,68) among GPs in undertaking oral cancer screening and prevention due to inadequate training (46–64%) (45). This lack of training was reiterated in qualitative studies:

 ‘I would be unhappy if [physicians] didn’t do a rectal exam. But I was not trained to routinely put my finger in someone’s mouth and feel around. I was trained to look’. (p. 375) (46)

Participants acknowledged that they learned ‘a bit [to examine the mouth] but there was little emphasis on cancers of the mouth and throat. The emphasis was on looking for swollen glands’. (p. 6) (51)

In some studies, GPs (91–100%) believed that dentists were more specialized than them to perform oral examinations (38,68). As one study highlighted:

‘It’s all down to the training of doctors and dentists, because dentists are the ones that know the mouth. They tend to know the mouth a lot better than the doctors because they’re seeing mouths every day. Doctors are looking at the whole body’. (p. 279) (68)

Some GPs felt that they could play a role in raising awareness about oral cancer particularly in patients with low health literacy who may often see a doctor first (46,51,60):

‘People associate dentists with teeth first and maybe gums. But when you talk about the tongue and buccal mucosa, they think of [a] doctor. The more educated might go to a dentist, but the average or poorly educated would probably seek out a physician’. (p. 375) (46)

GPs also felt that socio-economically disadvantaged patients may wait to seek oral health care resulting in delayed diagnosis, further reinforcing the crucial role they could play (60). In the context of the role of GPs in oral cancer prevention, some participants were also interested in receiving more information (46,51) on this topic:

‘It’s an important topic. . . I would like to see CME on that—maybe not a whole course, but as part of course on primary care review’. (p. 375) (46)

#### Domain 3: oral cancer practices

A total of 19 studies explored GPs’ oral cancer diagnostic and clinical practices. Ten studies (19,40,41,45,47,49,61,65,67) highlighted significant variability (7–70%) in routine oral check-up/screening practices among GPs. Such findings were also evident in two qualitative studies:

‘If the problem is below the neck, I rarely check for oral cancer’. (p. 6) (51)

‘Almost never do I spend much time looking [in the mouth] unless there is a complaint...’. (p. 375) (46)

Two studies reported that oral cavity examination was conducted by GPs only in case of complaints of soreness (94%) and prior history of pre-existing oral condition (81%) (60,68), while four studies reported that between 15% and 50% of GPs undertook oral cavity examination in older-aged patients (41,47,50,67). In four studies, 31.5–81% of GPs reported never conducting a routine oral cancer examination (40,48,66,67). Macpherson *et al*. (68) and Sohn *et al*. (50) identified lack of training (64–70%) and lack of time (15–47%) as key barriers in undertaking routine oral cancer examination by GPs. This was also reflected in the qualitative findings:

‘I do not recall having been taught how to perform an oral exam in any moment’ (p. 4) (60)

‘I think it’s a time issue. Ideally, we’d like to do it, but we don’t have the time or the resources’. (p. 280) (68)

Despite differences in oral cancer screening practices, 82.5–90% of GPs reported asking patients about risk practices, including alcohol and tobacco use while taking their medical history (38,41,47,48,65,68). However, counselling and educating patients regarding ill effects of risk habits of tobacco and alcohol use were not consistent (5–87%) among the GPs (40,48,51,65).

Several studies explored GPs’ referral practices for patients with oral cancer (40,46,61,64). GPs usually preferred to refer oral cancer cases to oral and maxillofacial surgeons (42–74.2%)(48, 61), followed by ear, nose and throat specialists (24–53%)(48, 61, 64) and dentists (9.3%) (64). This was also reflected in qualitative findings by Canto et al:

‘If I see leukoplakia or [other] suspicious lesion, I send [the patient] to [an] ENT first for biopsy ... [I]Rarely start with an oral surgeon’. (p. 375) (46)

## Discussion

This is the first integrative review to identify and appraise the research literature on the knowledge, attitudes and practices of GPs regarding oral cancer in developed countries. The quality of included studies was varied and there was diversity in designs, samples and results. The majority of studies were conducted in the USA, UK and Europe reflecting the changing trends of oral cancer incidence globally (23).

Overall, this review revealed limited oral cancer-related knowledge of GPs in developed countries. They had little information about emerging causative factors, including viral infections (48,63,65,68) and betel nut/betel quid use (40,41,63,64). An important finding from this review was the mixed understanding of GPs relating to the importance of early diagnosis of oral cancer for its prevention and treatment. These findings are perhaps not surprising given a lack of awareness among undergraduate medical students concerning oral cancer risk factors (57), suggesting there is not much information on oral cancer and associated facts in medical curricula. In addition, this inadequate oral cancer knowledge among GPs could also be a result of limited information gained through sources, such as scientific journals and continuing education courses (41,50,62,64). Given the increasing numbers of people migrating from developing countries where betel nut/betel quid chewing is endemic, GPs in developed countries will increasingly play an important role in preventing oral cancer through early detection. This will require education and awareness campaigns (34) that address both traditional and emerging oral cancer risk factors (23,69). This review also supports the suggestions of the inclusion of oral health education in the undergraduate medical curriculum (70) and implementation of continuing education courses (71) for GPs in order to recognize oral premalignant and malignant lesions to aid in obtaining an early diagnosis of oral cancer.

This review identified positive attitudes of GPs regarding their role in oral cancer prevention. However, their attitude about oral cancer-related knowledge was unclear as only a few of the GPs believed their knowledge to be current and updated (38,40,45). This belief of being equipped with limited knowledge could have played a role in shaping their self-confidence regarding oral cancer clinical practices. This review echoes the exploration by Wade *et al*. (72) regarding the influence of attitude on GPs’ intention to perform oral cancer examination. These findings are also consistent with a review by Florian *et al*. (73), which highlighted the mixed attitudes of GPs about facilitating discussions about risk factors with routine patients and subsequently suggested that identification of specific beliefs underlying such attitudes is essential to influence judgements of GPs. The findings from this review indicate the need for further oral cancer-related training for GPs (72) to enhance their confidence and comfort to do oral cancer screening and formulation of a universal approach to facilitate patient counselling (34) regarding the common, as well as emerging, risk factors like betel nut use.

It was evident that the knowledge and attitude of GPs towards oral cancer had an influence on their practices in this area. Their unclear attitude with limited oral cancer knowledge and training came up as a major deciding factor behind their practice of not conducting routine oral cancer screening until the patient complains (50,68). These findings echo the inadequate training of GPs regarding oral cavity examinations reported in previous literature (72,74). This review supports oral cancer screening in the medical curriculum (74) to increase the confidence of GPs to promote oral health.

The exact relationship of the length of experience in general practice with practitioners’ knowledge, confidence and intention to practice oral cancer screening procedures could not be assessed through this review since a very limited number of studies reported this link (38,47,65); this area needs to be explored through further research. This review also indicated the lack of clear oral cancer-related referral guidelines for GPs (40,46,61,64) as the differences were apparent in their opinions for the preferred specialty for suspected oral cancer cases (48,61,64). These findings complement the previous literature (75–77), which highlights gaps in oral cancer referral systems and unclear guidelines regarding referrals. Interestingly though, the UK does have oral cancer referral guidelines requiring GPs to refer patients first to dentists for further assessment (78,79). However, these guidelines have been challenged by researchers due to the lack of accessible and affordable dental referral pathways for patients (80–82) in the UK, which could lead to further delays in diagnosis. Further, a recent systematic review of patient journeys in the diagnosis of oral cancer found no evidence to suggest that GPs performed less well than dentists in terms of referrals to specialists (81). Our findings along with previous research (75,81) suggest the need to also design and include a standard referral pathway globally for definitive care and management of oral cancer to reduce any further delays in initializing the treatment.

### Implications of the findings

The findings from this review have significant implications for training, clinical practice and research. The inclusion of specific education in the medical curriculum for a better understanding of oral cancer causative factors and pathogenesis could be beneficial. Although the general practice is already overburdened, further oral cancer-related training (e.g. online resources and continuing education courses) aimed at GPs could be undertaken to help them in identifying signs and symptoms of oral cancer. The short training modules for medical practitioners regarding emerging oral cancer risk factors like betel nut are needed considering the changing migration patterns and oral cancer trends in the world.

Furthermore, strategies to encourage the prompt identification of oral cancer through opportunistic screening of high-risk patients (e.g. those >45-years old, who engage in tobacco and/or alcohol consumption over the recommended limits or chew betel/areca nut (69,83)) could assist GPs to improve the poor oral cancer survival rates. Likewise, routine visual inspection of the oral mucosa of patients (using a torch and dental mirrors) can be incorporated in general practice as a more feasible and affordable method than expensive dental check-ups for early diagnosis of oral malignancies. Moreover, GPs could also be motivated to provide one-to-one health advice to high-risk patients during risk factor counselling (such as tobacco and alcohol cessation), which can be effective if tailored to individual needs and circumstances.

Lastly, in light of the limited number of studies in this area, future research regarding oral cancer-related practices of GPs must be undertaken in other developed countries like Australia and Canada, where there has been a great influx of South Asian immigrants in recent years, particularly from India.

### Limitations

The literature search for this review was limited to four databases and did not include any grey literature nor unpublished studies or articles in other languages. Therefore, all studies in this area may have not been retrieved in the literature search. The diversity in methodology and quality of included studies may have compromised the reliability of the findings. The studies reviewed were undertaken in the context of oral cancer only; hence, the findings cannot be generalized to other cancers. Additionally, the review was limited to studies conducted in developed countries and the findings may not apply to GPs practicing in developing countries, particularly those with high rates of oral cancer.

## Conclusion

This integrative review is first of its kind to provide valuable insight into GPs’ perspectives and clinical practices regarding oral cancer. The pivotal role of GPs in developed countries is universally seen as the first point of contact in primary health care and a gateway to access secondary health care services. However, this review has identified gaps in their oral cancer-related knowledge, attitude towards oral cancer risk and screening practices. The limited knowledge of GPs is apparent as they are not updated regarding emerging oral cancer risk factors like betel nut/quid use and identification techniques to detect oral malignancy. Furthermore, GPs present mixed attitudes with inconsistent clinical practices relating to routine oral cancer screening, patient counselling and referrals, which is concerning for oral cancer prevention. These findings suggest the need for further education and training of GPs regarding timely diagnosis and referral of oral cancer cases in association with patient guidance to promote oral cancer awareness.

## Supplementary material

Additional file 1: Example of search strategy

Additional file 2: Full text screening (table)

Additional file 3: Critical appraisal of articles (table)

Additional file 4: Data extraction tool

## Declarations

Funding: not applicable.

Ethical approval: not applicable.

Conflicts of interest: the authors declare that they have no conflict of interest.

## Supplementary Material

cmaa026_suppl_Supplementary_Additional_File_01

cmaa026_suppl_Supplementary_Additional_File_02

cmaa026_suppl_Supplementary_Additional_File_03

cmaa026_suppl_Supplementary_Additional_File_04
