# Supplementary figures and images for: Knowledge, attitudes and practices of general medical practitioners in developed countries regarding oral cancer: an integrative review
Source: Fam Pract. 2020 Apr 7;37(5):592–605. doi: 10.1093/fampra/cmaa026 (PMC7759340; doi:10.1093/fampra/cmaa026)

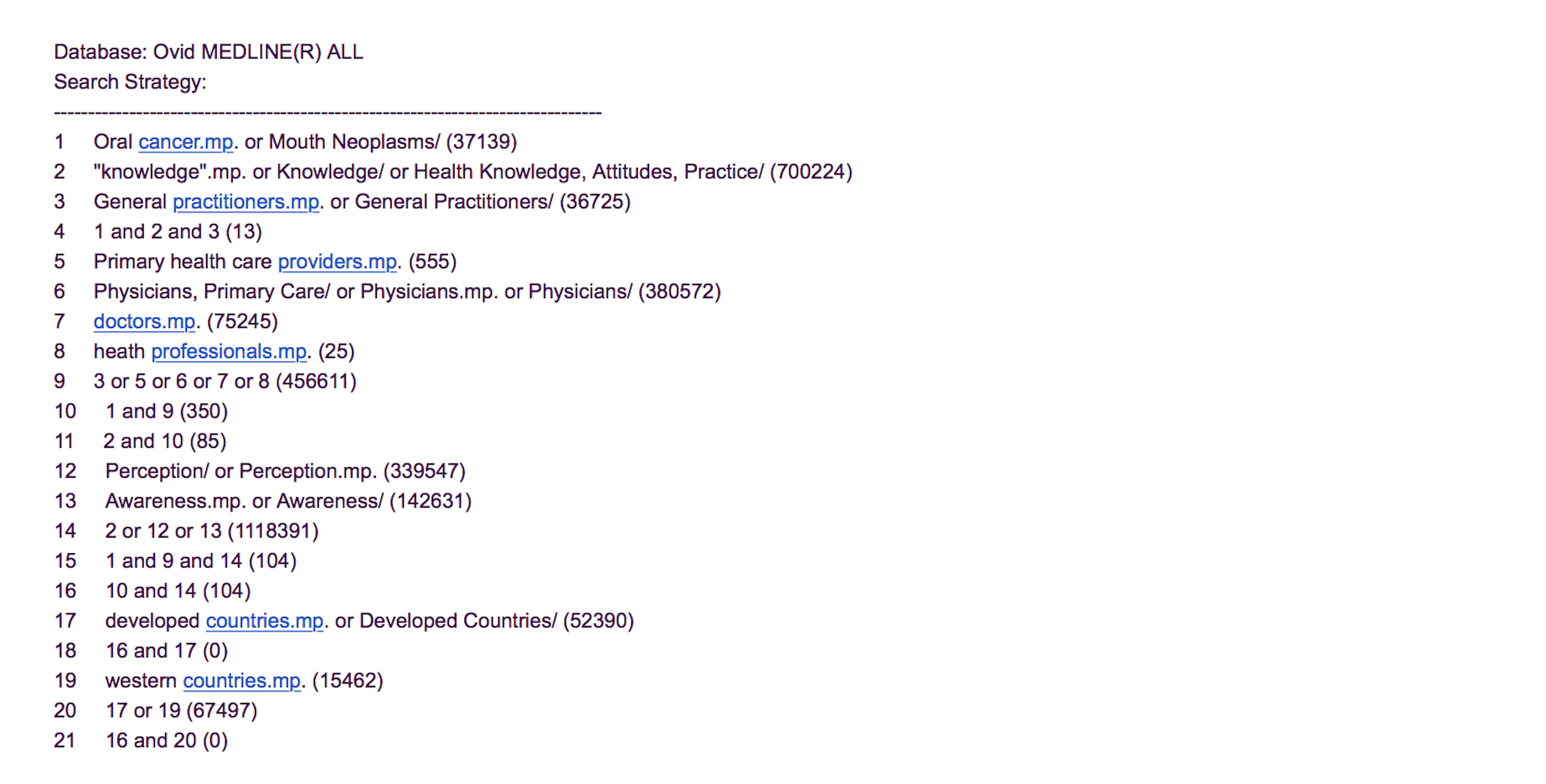

Supplement: cmaa026_suppl_Supplementary_Additional_File_01 [file cmaa026_suppl_supplementary_additional_file_01.png]
