# Supplementary material for: Knowledge, attitudes and practices of general medical practitioners in developed countries regarding oral cancer: an integrative review
Source: Fam Pract. 2020 Apr 7;37(5):592–605. doi: 10.1093/fampra/cmaa026 (PMC7759340; doi:10.1093/fampra/cmaa026)
Supplement: cmaa026_suppl_Supplementary_Additional_File_02 [file cmaa026_suppl_supplementary_additional_file_02.docx]

Full text Screening of Articles

| S.No. | Title  - (Author, Year)  -Study design | Screening status | Explanation | Database/Reviewer |
| --- | --- | --- | --- | --- |
| 1 | Oral cancer knowledge, attitudes  and practices A survey of dentists and primary care physicians in Massachusetts  (Applebaum et al., 2009)  Cross sectional questionnaire | Included | The authors conducted a study to assess dentists’ and primary care physicians’ oral cancer knowledge, attitudes and practices in the Commonwealth of Massachusetts. | CINAHL/NS |
| 2 | Oral Cancer Preventive Practices of South Carolina Dentists and Physicians  (Reed et al., 2010)  Cross sectional questionnaire | Included | Assessment of the South Carolina (SC) dentists’ and physicians’ oral cancer knowledge and practices and interest in training in oral cancer screening and tobacco cessation counselling. | CINAHL/NS |
| 3 | Diagnosing and treating common oral pathologies  (Dilley et al., 1991) | Excluded | Literature review regarding most common oral pathologies that might be seen in a paediatric practice. | OVID/NS |
| 4 | Health professionals' baseline knowledge of oral/pharyngeal cancers  (McCunniff et al, 2000)  Cross sectional questionnaire | Included | The purpose of this study was to evaluate: self-perceived competency of Health Professionals in screening for oral/pharyngeal cancers (OPCs); knowledge about their signs and risk factors. | OVID/NS |
| 5 | Possibilities of preventing osteoradionecrosis during complex therapy of tumours of the oral cavity  (Nemeth et al, 2000) | Excluded | This article doesn’t focus on oral cancer related Knowledge, attitudes and practices (Related to osteoradionecrosis during treatment) | OVID/NS |
| 6 | Professional and community efforts to prevent morbidity and mortality from oral cancer  (Alfano et al, 2001) | Excluded | Literature review. The authors summarize several approaches to educating and mobilizing the dental profession and the public about oral cancers. | OVID/NS |
| 7 | Survey of hospital doctors’ attitudes and knowledge of oral conditions in older patients  (Morgan et al, 2001) | Excluded | Nothing specifically about Knowledge, attitudes and practices of GMPs about oral cancer. Study was designed to assess the views and knowledge of hospital doctors in general and geriatric medicine on oral health in older people. | OVID/NS |
| 8 | The role of primary healthcare professionals in oral cancer prevention and detection  (Macpherson et al, 2003)  Mixed method(Questionnaire + Focus group + Interview) | Included | Study to investigate current knowledge, examination habits and preventive practices of primary healthcare professionals in Scotland, with respect to oral cancer, and to determine any relevant training needs. | OVID/NS |
| 9 | Knowledge of oral cancer and screening practices of primary care providers at Federally Qualified Health Centres  (Sohn et al, 2005)  Mixed method (Questionnaire +Focus group) | Included | Primary care providers (PCPs) who worked in Federally-Qualified Health Centres (FQHC) in Michigan were surveyed to assess their knowledge level and practices related to screening and preventing oral cancer. | OVID/NS |
| 10 | Picture guide--diagnosing oral cancer  (Joshi et al, 2006) | Excluded | Literature Review article | OVID/NS |
| 11 | Adequacy of training in oral cancer prevention and screening as self-assessed by physicians, nurse practitioners, and dental health professionals  (Patton et al, 2006)  Cross sectional survey | Included | Self-reported adequacy of training (Practice) in OPC early intervention activities was compared among 4 health care provider groups in North Carolina. | OVID/NS |
| 12 | Oral cancer awareness of general medical and general dental practitioners  (Carter et al, 2007)  Cross sectional Survey | Included | Assessment of general medical practitioners’ (GMPs’) and general dental practitioners’ (GDPs’) awareness of prevention and early detection of oral cancer. | OVID/NS |
| 13 | The Cultural and Social Context of Oral and Pharyngeal Cancer Risk and Control among Hispanics in New York  (Cruz et al, 2007)  Qualitative (Focus groups + Interviews) | Included | This qualitative assessment explores OPC awareness, attitudes, and screening practices among at-risk Hispanics, health care providers, and community leaders in a Hispanic neighbourhood of NYC.  Hispanics only | OVID/NS |
| 14 | Accuracy of referrals to a specialist oral medicine unit by general medical and dental practitioners and the educational implications  (Sardella et al, 2007) | Excluded | Nothing specifically about Knowledge, attitudes and practices of GMPs about oral cancer. Investigated the accuracy of diagnoses of oral mucosal diseases made by family physicians (compared referral letters) | OVID/NS |
| 15 | Training in the primary prevention and early detection of oral cancer: pilot study of its impact on clinicians' perceptions and intentions  (LeHew et al, 2009)  Interventional study (Pre-intervention survey) | Excluded | Data regarding GMPs could not be elicited because of multiple healthcare provider. This pilot study tested a new program for training medical care providers in the primary prevention and early detection of oral cancer. | OVID/NS |
| 16 | Oral cancer--current knowledge, practices and implications for training among an Irish general medical practitioner cohort  (Ni Riordain et al, 2009)  Cross sectional Survey | Included | Study investigated the current knowledge and practices of general medical practitioners (GMPs) in Ireland regarding the examination of the oral cavity and the detection of oral malignancy and the training they had received at both undergraduate and postgraduate level and since commencing in practice | OVID/NS |
| 17 | Why must physicians know about oral diseases?  (Ramirez et al, 2010) | Excluded | Literature Review. (Medical practitioners must play an active role in oral health promotion.) | OVID/NS |
| 18 | Health professional's perceptions of and potential barriers to smoking cessation care: a survey study at a dental school hospital in Japan  (Saito et al, 2010) | Excluded | Nothing specifically about Knowledge, attitudes and practices of GMPs about oral cancer.  (questions about smoking cessation rather than oral cancer) | OVID/NS |
| 19 | Early diagnosis in primary oral cancer: is it possible?  (Van der Waal, 2011) | Excluded | Literature Review. Study to explain that the delay in diagnosis of oral cancer is caused both by patients' delay and doctors' delay. | OVID/NS |
| 20 | A marketing campaign to promote screening for oral cancer  (Ismail et al, 2012)  Interventional study (Pre-intervention survey) | Included | The authors present data about the effectiveness of the campaign with regard to the oral cancer screening behaviours/practices of medical and dental providers. | OVID/NS |
| 21 | Perspectives of San Juan healthcare practitioners on the detection deficit in oral premalignant and early cancers in Puerto Rico: a qualitative research study  (Morse et al, 2011) | Included | Study regarding perspectives of San Juan healthcare practitioners whose practice could be involved in the detection of oral lesions. | OVID/NS |
| 22 | Outcomes of oral cancer early detection and prevention statewide model in Maryland  (Maybury et al, 2012) | Excluded | Literature Review. High oral cancer incidence rate prompted Maryland to develop a state wide approach to oral cancer early detection and prevention. | OVID/NS |
| 23 | Knowledge of diagnostic and risk factors in oral cancer: results from a large-scale survey among non-dental healthcare providers in Northern Germany  (Hertrampf et al, 2014)  Cross sectional Survey | Included | Reports knowledge of GMPs about oral cancer. A self-administered questionnaire regarding oral cancer was mailed to physicians from various fields, such as otorhinolaryngology, general practice, internal medicine, and dermatology. | OVID/NS |
| 24 | Information about oral cancer on the Internet: our patients cannot understand it  (Varela-Centelles et al, 2015) | Excluded | Nothing specifically about Knowledge, attitudes and practices of GMPs about oral cancer. Used 3 search engines to select websites on oral cancer then assessed their readability | OVID/NS |
| 25 | Current training provision and training needs in oral health for UK general practice trainees: survey of General Practitioner Training Programme Directors  (Ahluwalia et al, 2016) | Excluded | Nothing specifically about Knowledge, attitudes and practices of GMPs about oral cancer. A cross-sectional survey of GP Training Programme Directors using an online questionnaire asking about current oral health education training | OVID/NS |
| 26 | Promoting oral cancer examinations to medical primary care providers: a cluster randomized trial  (Wee et al, 2016)  RCT | Excluded | Nothing specifically about Knowledge, attitudes and practices of GMPs about oral cancer. (To compare the percentage of patients who had an oral cancer examination (OCE) by their primary care provider (PCP) in medical clinics. – Intervention study) | OVID/NS |
| 27 | Evaluating a Web-Based Educational Module on Oral Cancer Examination Based on a Behavioral Framework  (Wee et al, 2016) | Excluded | Nothing specifically about Knowledge, attitudes and practices of GMPs about oral cancer. Aims to design a web-based educational program based on a behavioural framework to encourage PCPs to conduct OCE. | OVID/NS |
| 28 | Epidemiology of primary oral cancer diagnostics in Kaunas  (Gelazius et al, 2018)  Cross sectional Survey | Included | To find out the insight of primary oral cancer diagnostics in Kaunas city. | OVID/NS |
| 29 | Knowledgeability, Attitude and Behavior of Primary Care Providers Towards Oral Cancer: A Pilot Study  (Shimpi et al, 2018)  Cross sectional Survey | Included | The objective of this study was to assess current knowledgeability, attitudes, and practice behaviours of primary care providers (PCPs) towards oral cancer screening | OVID/NS |
| 30 | A primary care study of dentists' and doctors' knowledge of oral cancer  (Fisher et al, 2001) | Excluded | To compare the knowledge of oral cancer and related issues of general dental and general medical practitioners (GDPs and GMPs). (reports/reviews another article by Greenwood et al) | ProQuest Central/NS |
| 31 | Primary care clinicians' knowledge of oral cancer: a study of dentists and doctors in the North East of England  (Greenwood et al, 2001)  Cross sectional Survey | Included | To compare the knowledge of oral cancer and related issues of general dental and general medical practitioners (GDPs and GMPs) | ProQuest Central/NS |
| 32 | Assessing physicians' and dentists' oral cancer knowledge, opinions and practices  (Yellowitz et al, 1995)  Cross sectional Survey | Included | This pilot project assessed the knowledge, opinions and practices of a group of Maryland physicians and dentists in oral cancer prevention, diagnosis and detection. | Scopus/NS |
| 33 | Views of oral cancer prevention and early detection: Maryland physicians  (Canto et al, 2002)  Qualitative (Focus group + Interviews) | Included | The purpose of this study was to obtain in-depth information on Maryland physicians' knowledge, opinions and practices about oral cancer examinations. | Scopus/NS |
| 34 | Maryland family physicians' knowledge, opinions and practices about oral cancer  (Canto et al, 2002) | Included | To assess family physicians' knowledge, opinions and practices regarding oral cancers in the state of Maryland, USA. | Scopus/NS |
| 35 | Knowledge and behaviours of primary care physicians on oral cancer in Italy  (Nicotera et al, 2004)  Cross sectional Survey | Included | Investigated the knowledge and behaviours of a random sample of 500 primary care physicians (PCPs) regarding oral cancer in Calabria (Italy). | Scopus/NS |
| 36 | Oral cancer awareness for the general practitioner: New approaches to patient care  (Farah et al, 2008) | Excluded | Nothing specifically about Knowledge, attitudes and practices of GMPs about oral cancer. Explore clinically available modalities that may be used by the general dental practitioner.  (clinically available techniques only) | Scopus/NS |
| 37 | Doctors' tacit knowledge on coping processes of oral cancer patients: A qualitative study  (Rana et al, 2016) | Excluded (psychological aspect only) | Nothing specifically about Knowledge, attitudes and practices of GMPs about oral cancer. Doctors working in the field of oral and maxillofacial surgery, otorhinolaryngology and oncology were interviewed about coping and quality of life of patients. | Scopus/NS |
| 38 | Raising awareness of oral cancer from a public and health professional perspective  (Macpherson et al, 2018) | Excluded | Literature Review. Overview of the evidence of effectiveness of interventions aimed at raising cancer awareness and explores the use of mass media for health behaviour change. | Scopus/NS |
| 39 | Oral cancer awareness of non-consultant hospital doctors in Irish hospitals  (Shanahan et al, 2018)  Cross sectional Survey | Included | The aim of this study is to assess the level of awareness of oral cancer amongst non-consultant hospital doctors (NCHDs) in Ireland. | Scopus/NS |
| 40 | Sources and patterns of referrals of oral cancer: role of general practitioners  (Scully et al, 1986) | Excluded | Nothing specifically about Knowledge, attitudes and practices of GMPs about oral cancer. Study emphasises the importance of educating patients about oral cancer. | Manual/NS |
| 41 | Oral cancer prevention: the role of family practitioners  (Goodman et al, 1995) | Excluded | Literature Review. To assess knowledge, opinions and practices of health care providers and public concerning oral cancer. | Manual/NS |
| 42 | Role of general practice in the diagnosis of oral cancer  (Crossman et al, 2016) | Excluded | Literature Review. To investigate the symptoms associated with cancer of the oral cavity and to explore the role of general practitioners (GP) in the identification and referral of patients. | Manual/NS |
| 43 | A cross-sectional survey of awareness of human papillomavirus-associated oropharyngeal cancers among general practitioners in the UK  (Lechner et al, 2018)  Cross sectional Survey | Included | Developed a short questionnaire among 340 GPs assessing demographic characteristics of the participants, self-rated knowledge of OPC, awareness of OPC risk factors, awareness of time trends in smoking-related and HPV-related OPC | Manual/NS |
