# Supplementary material for: Knowledge, attitudes and practices of general medical practitioners in developed countries regarding oral cancer: an integrative review
Source: Fam Pract. 2020 Apr 7;37(5):592–605. doi: 10.1093/fampra/cmaa026 (PMC7759340; doi:10.1093/fampra/cmaa026)
Supplement: cmaa026_suppl_Supplementary_Additional_File_03 [file cmaa026_suppl_supplementary_additional_file_03.docx]

| Additional file 3: Critical Appraisal of Articles | | | | | |
| --- | --- | --- | --- | --- | --- |
| S.No. | Author  Year of publication | Methodology | Score/total items | Percentage (in %) | Quality |
| 1 | Yellowitz et al  1995 | Quantitative | 6/8 | 75 | B |
| 2 | McCunniff  2000 | Quantitative | 4/8 | 50 | B |
| 3 | Greenwood et al  2001 | Quantitative | 4/8 | 50 | B |
| 4 | Canto et al  2002 | Quantitative | 3/8 | 37.5 | C |
| 5 | Canto et al  2002 | Qualitative | 8/10 | 80 | A |
| 6 | Macpherson et al  2003 | Mixed method | 3/8 | 37.5 | C |
| 7 | Nicotera et al  2003 | Quantitative | 4/8 | 50 | B |
| 8 | Sohn et al  2005 | Quantitative | 4/8 | 50 | B |
| 8 | Patton et al  2006 | Quantitative | 5/8 | 62.5 | B |
| 10 | Cruz et al  2007 | Qualitative | 8/10 | 80 | A |
| 11 | Carter et al  2007 | Quantitative | 2/8 | 25 | C |
| 12 | NiRiordain et al  2009 | Quantitative | 3/8 | 37.5 | C |
| 13 | Applebaum et al  2009 | Quantitative | 5/8 | 62.5 | B |
| 14 | Reed et al  2010 | Quantitative | 4/8 | 50 | B |
| 15 | Morse et al  2011 | Quantitative | 9/10 | 90 | A |
| 16 | Ismail et al  2012 | Mixed method | 6/8 | 75 | B |
| 17 | Hertrampf et al  2014 | Quantitative | 5/8 | 62.5 | B |
| 18 | Shanahan et al  2018 | Quantitative | 4/8 | 50 | B |
| 19 | Shimpi et al  2018 | Quantitative | 5/8 | 62.5 | B |
| 20 | Gelazius et al  2018 | Quantitative | 2/8 | 25 | C |
| 21 | Lechner et al  2018 | Quantitative | 4/8 | 50 | B |

Note: A or good quality (80-100%); B or fair quality (50-79%); C or poor quality (<50%)

Reference: Goldsmith MR, Bankhead CR, Austoker J. Synthesising quantitative and qualitative research in evidence-based patient information. Journal of Epidemiology & Community Health. 2007;61(3)
