# Supplementary material for: Knowledge, attitudes and practices of general medical practitioners in developed countries regarding oral cancer: an integrative review
Source: Fam Pract. 2020 Apr 7;37(5):592–605. doi: 10.1093/fampra/cmaa026 (PMC7759340; doi:10.1093/fampra/cmaa026)
Supplement: cmaa026_suppl_Supplementary_Additional_File_04 [file cmaa026_suppl_supplementary_additional_file_04.docx]

| **Author** |  | | | |
| --- | --- | --- | --- | --- |
| **Year** |  | | | |
| **Country** |  | | | |
| **Title** |  | | | |
| **Sample size, age and methodolgy employed** |  | |  |  |
| **Years of experience/years after graduation and Response Rate** |  | | |  |
| **Method of data collection** | **Questionnaire, Interviews, Focus groups** | | |  |
| **Study Outcomes** | **Knowledge, Attitude and Practices in relating to Oral cancer** | | |  |
| **1.Knowledge** | ☐✓  ☐✗ | 1) Level of information about oral cancer risk factors………  2) Familiarity of most common sites and symptoms of oral cancer  3) Awareness regarding oral cancer diagnosis and treatment strategies.  4) Source of information on OH Knowledge:  5) Other key findings …………………… | |  |
| **2. Attitudes** | ☐✓  ☐✗ | 1) Perception on oral cancer knowledge: ………....................  2) Perceived importance of oral cancer identification………………….  3) Inclination towards oral cancer prevention……………………………  4) Other key findings………………………………… | |  |
| **3. Practices** | ☐✓  ☐✗ | 1) oral cancer diagnostic practices=…………  2) Oral cancer routine screening practices: …………….  3) Patient counselling regarding risk factors: …………….  4) oral cancer-related referral practices  5) Other key findings | |  |

**Additional file 4: Data Extraction Form**
